# Emergency transportation interventions for reducing adverse pregnancy outcomes in low- and middle-income countries: a systematic review protocol

**DOI:** 10.1186/s13643-018-0729-2

**Published:** 2018-04-25

**Authors:** John Ehiri, Halimatou Alaofè, Ibitola Asaolu, Joy Chebet, Ekpereonne Esu, Martin Meremikwu

**Affiliations:** 1 Department of Health Promotion Sciences, Mel and Enid Zuckerman College of Public Health, University of Arizona, 1295 N. Martin Avenue, Tucson, AZ 85724 USA; 2 Department of Public Health, University of Calabar Teaching Hospital, Moore Road, Calabar, Cross River State Nigeria

**Keywords:** Emergency obstetric transportation, Maternal, child, and newborn health, Obstetric complications, Three-delay framework, Low- and middle-income countries, Maternal health, Newborn health, Child health, Global maternal and child health

## Abstract

**Background:**

Transportation interventions seek to decrease delay in reaching a health facility for emergency obstetric care and are, thus, believed to contribute to reductions in such adverse pregnancy and childbirth outcomes as maternal deaths, stillbirths, and neonatal mortality in low- and middle-income countries (LMICs). However, there is limited empirical evidence to support this hypothesis. The objective of the proposed review is to summarize and critically appraise evidence regarding the effect of emergency transportation interventions on outcomes of labor and delivery in LMICs.

**Methods:**

The following databases will be searched from inception to March 31, 2018: MEDLINE/PubMed, EMBASE, Web of Science, EBSCO (PsycINFO and CINAHL), the Cochrane Pregnancy and Child Birth Group’s Specialized Register, and the Cochrane Central Register of Controlled Trials. We will search for studies in the grey literature through Google and Google Scholar. We will solicit unpublished reports from such relevant agencies as United Nations Fund for Population Activities (UNFPA), the World Health Organization (WHO), the United Nations Children’s Fund (UNICEF), the United States Agency for International Development (USAID), and the United Kingdom Department for International Development (DfID) among others. Data generated from the search will be managed using Endnote Version 7. We will perform quantitative data synthesis if studies are homogenous in characteristics and provide adequate outcome data for meta-analysis. Otherwise, data will be synthesized, using the narrative synthesis approach.

**Discussion:**

Among the many barriers that women in LMICs face in accessing life-saving interventions during labor and delivery, lack of access to emergency transportation is particularly important. This review will provide a critical summary of evidence regarding the impact of transportation interventions on outcomes of pregnancy and childbirth in LMICs.

**Systematic review registration:**

PROSPERO CRD42017080092

**Electronic supplementary material:**

The online version of this article (10.1186/s13643-018-0729-2) contains supplementary material, which is available to authorized users.

## Background

Globally, thousands of mothers, newborns, and children in low-income countries die from pregnancy and childbirth-related complications annually. Additionally, millions suffer various debilitating consequences of pregnancy. In 2015, an estimated 303,000 women died from pregnancy and childbirth-related complications [1, 2], primarily in low- and middle-income countries (LMICs). At an estimated 546 maternal deaths per 100,000 live births [3], sub-Saharan Africa (SSA) has the highest maternal mortality ratio (MMR) of all world regions. Although significant progress was made in achieving the Millennium Development Goal (MDG) 5 of decreasing global MMR from 385 to 216 maternal deaths per 100,000 live births, this reduction fell short of the target 75% decrease. Similarly, child mortality rate decreased. Under-five mortality declined by 49% between 1990 and 2015, but still fell significantly short of the MDG-4 target of a two-third reduction [4]. In 2016, under-five mortality was 5.6 million [5]. Neonates accounted for 45% of under-five deaths [4], and this share exceeded 50% in several regions [5]. A significant proportion of under-five deaths happen during the perinatal period. For example, preterm birth and intrapartum-related complications account for 59% of all newborn deaths [4], and in 2015, over 45% of the 2.6 million stillbirths occurred during the intrapartum period [6, 7].

Although cost-effective interventions can be implemented to reduce these appalling statistics [8, 9], high coverage of essential interventions within healthcare facilities does not necessarily improve maternal, newborn, and child health (MNCH) outcomes [10], largely because of inaccessibility of services. Indeed, each year, there are around 136 million births, of which around 60 million occur outside health facilities [11]. During an obstetric emergency, every moment of delay in seeking and receiving skilled obstetric care increases the risks of stillbirth, neonatal, or maternal death. For some emergencies such as antepartum hemorrhage, even hours can be the difference between life and death for the mother and the fetus. Many of the estimated 1.4 million intrapartum stillbirths and 1.5 million early neonatal deaths could be avoided with access to skilled care at birth, timely emergency obstetric care, and immediate newborn care [12]. Unfortunately, skilled attendance at birth remains unacceptably low at 52% in sub-Saharan Africa [11], suggesting a major gap that warrants intervention during childbirth and the perinatal period.

### Description of the intervention and how it might work

A functioning continuum of care between the home, primary health care centers, and secondary healthcare facilities where emergency obstetric care can be provided is required to link women and newborns to quality care with minimum delay [13]. Current literature suggests that delays in reaching healthcare facilities for emergency obstetric care in LMICs can be reduced through implementation of transportation programs [14]. Transportation interventions for emergency obstetric care include all financing schemes that enable poor pregnant women to overcome barriers of transportation to health facilities for emergency obstetric care during labor and delivery. Such interventions include direct provision of transportation for pregnant women in need of emergency obstetric care in an appropriate healthcare facility. Examples include the provision of motorbike ambulances specially engineered for use in rough terrains in resource-poor communities [15]. Currently, several non-governmental organizations (NGOs) are engaged in the provision of emergency obstetric transportation in many LMICs, using motorbike ambulances, bicycle ambulances, cycle rickshaws, wheeled stretchers, canoes, and ox carts [16–18]. Many causes of maternal mortality such as severe bleeding after childbirth, post-delivery infections, obstructed labor, and blood pressure disorders are preventable or treatable conditions [19]. While most women would have normal pregnancies and safe deliveries, unanticipated obstetric complications and emergencies sometimes occur. In resource-poor settings where many women deliver at home or in inadequately equipped health centers, ensuring that those who develop obstetric emergencies during childbirth are quickly transported to facilities where they can receive quality emergency obstetric care can be the difference between life and death for the pregnant woman and her fetus. Unfortunately, referral to needed emergency obstetric care may not be possible for a plethora of reasons, including geography, cost, and lack of transportation. Transportation interventions seek to decrease delay in reaching a health facility for emergency obstetric care and are, thus, believed to contribute to reductions in such adverse pregnancy and birth outcomes as maternal deaths, stillbirths, and neonatal mortality in low- and middle-income countries (LMICs) [20].

### Conceptual framework for the review

**Fig. 1:**
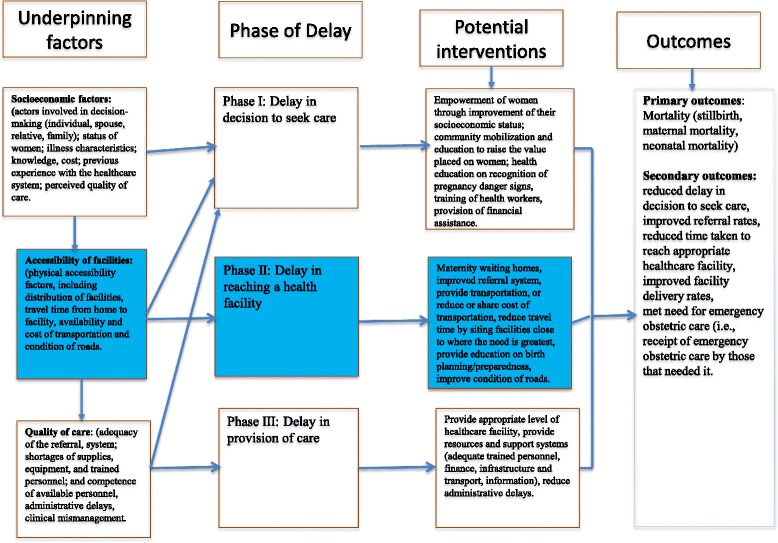
Conceptual framework of the review

Many barriers in access to care exist, particularly for women in rural settings in LMICs [21, 22]. The conceptual model for this review (Fig. 1) is based on the three-delay framework for analyzing maternal mortality in low- and middle-income countries [23]. Phase 1 delay refers to delays in the recognition of potentially life-threatening complications/emergencies and the decision to seek care at a healthcare facility, phase II refers to delays in time to reach a healthcare facility, and phase III delay refers to delays in receiving care once a woman reaches a healthcare facility [23–25]. An audit of perinatal deaths that occurred in a regional hospital in western Tanzanian between 2002 and 2004 revealed that phase I delays occurred in 19% of the cases, phase II delays in 21.5% of the cases, and phase III delays in 72.5% [26]. Many social factors influence the decision to seek care, including lack of knowledge about the seriousness of complications, not knowing where to receive care, and waiting to receive permission from the husband or other family decision-makers [27]. Furthermore, an analysis of Demography and Health Survey (DHS) data from 41 countries showed that the most common obstacles to seeking obstetric care were financial barriers (> 50%), challenges with transportation (37%), and distance (37%) [28]. Lack and high costs of transportation, poor road conditions, and time to arrange transport may also increase the time to reach a health facility [29–31]. Emergency obstetric transportation interventions are designed to address phase II delays, i.e., delays occur after the decision to seek care is made and before obtaining obstetric care. Thus, this review will focus on assessment of the effects of emergency transportation interventions that were implemented to address phase II delays aimed at reducing adverse pregnancy and birth outcomes in LMICs.

Faced with the challenge of unacceptably high maternal mortality rates, community organizations in many LMICs mobilize to provide free emergency obstetric transportation for pregnant women in need. For example, in several communities in northern Nigeria where the maternal mortality ratio is more than twice the national average [32, 33], the National Union of Road Transport Workers (NURTW) in conjunction with the Amalgamated Commercial Motorcycle Riders Association of Nigeria (ACOMORAN) operate a scheme to provide emergency transportation for pregnant women. Other types of obstetric emergency transportation schemes include community health insurance and pre-payments, conditional cash transfers, vouchers, loans, and revolving funds aimed at alleviating the cost of transportation to needed emergency obstetric care [15, 33–37]. In Kenya for example, the Maternal and Newborn Improvement (MANI) project uses a transport voucher to assist poor pregnant women to access health services [38]. In this project, community health workers register local transport providers and identify poor women who will receive transport vouchers. When due to deliver, the pregnant woman contacts a registered driver who takes her to the nearest health facility. On verification of the transaction, the project sends a mobile money transfer payment to the transporter. Emergency obstetric transportation programs have also been described in parts of Asia. Por et al. [39] describe a voucher scheme that is designed to increase access to skilled birth attendants for poor women in three rural health districts in Cambodia. With funding support from the Belgian Technical Cooperation, the Cambodian Ministry of Health established a voucher scheme and other financial incentives in 2007 that were aimed at improving access to safe delivery for poor women in Cheung Prey and Chamkar districts of Kampong Cham province. Evaluation of this program shows that the scheme increased facility-based deliveries from 16.3 to 44.9% over a 2-year period. This marked increase in skilled birth attendance was attributed to reduced financial burden on families [39, 40]. Similar maternal voucher schemes aimed at reducing transport barriers for poor pregnant women have been described in Bangladesh [41] and in Pakistan [42].

### Why it is important to conduct this review

Although many obstetric emergency transportation interventions are being implemented in LMICs, there is limited empirical evidence to show their effectiveness in reducing adverse outcomes associated with labor and delivery. The only available study on the subject is a 2015 systematic review that focused on only community-based loan funds for transportation during obstetric emergencies in developing countries [43]. This review demonstrated that compared to women in the control communities, those in sites where community-based loan funds were implemented experienced higher reductions in maternal mortality, had higher rate of facility-based deliveries, and had increased utilization emergency obstetric care. This review is limited in scope, given that loan funds represent only one type of intervention that can be implemented to reduce the financial barriers that women in low- and middle-income countries face in accessing transportation for obstetric care. To increase the scope of available evidence, this review will summarize and critically appraise available data on the effect of all forms of interventions and financing mechanisms to promote transportation for emergency obstetric care in LMICs. It is hoped that the findings will inform the global debate on access to routine and emergency obstetric services in LMICs.

## Methods

This protocol is registered in PROSPERO (ID: CRD42017080092) and will follow the Preferred Reporting Items for Systematic Review and Meta-Analysis Protocols (PRISMA-P) guidelines (Additional file 1).

### Inclusion criteria

#### Types of studies

Quasi-randomized controlled and randomized controlled trials that assessed the effect of transportation interventions on pregnancy outcomes in LMICs will be included. If there is paucity of controlled experimental studies, we will include controlled before-and-after studies and cohort studies with control.

#### Study population

The study population consists of women in the prenatal, intrapartum, or post-natal phase of pregnancy with an obstetric complication, who were referred from the community or from a primary health care center to a higher-level facility that can provide emergency obstetric care.

#### Types of intervention

The types of intervention consist of all financing schemes or in-kind initiatives that enable poor pregnant women to overcome barriers of transportation to health facilities for emergency obstetric care in the prenatal period, during labor, delivery, or up to 42 days after delivery (postpartum period). Such interventions include programs that provide ground or water transportation (e.g., bicycle, motorcycle, oxcart, ambulance, boats) where none existed prior or that provide vouchers/subsidies, loans, or sets up a system of pooled funds or in-kind support for transportation to health facilities for needed emergency obstetric care during labor, delivery, or within the postpartum period.

#### Type of comparison

No transportation intervention for emergency obstetric care.

#### Type of outcomes

Primary outcomes will encompass mortality (stillbirth, maternal mortality, and neonatal mortality). Secondary outcomes will include reduced delay in decision to seek care, improved referral rates, reduced time taken to reach appropriate healthcare facility, improved facility delivery rates, and met need for emergency obstetric care (i.e., receipt of emergency obstetric care by those that needed it).

#### Context

This review will be restricted to studies conducted in countries designated as low and middle income according to the World Bank’s classification [44].

### Exclusion criteria

This review will exclude (1) studies conducted outside LMICs, (2) observational studies without a control arm, and (3) interventions in refugee camps and conflict-affected settings.

### Search strategy

We will search the following databases from inception to March 31, 2018: MEDLINE/PubMed, EMBASE, Web of Science, EBSCO (PsycINFO and CINAHL), SCIELO, LILACS, JSTOR, POPLINE, the Cochrane Pregnancy and Child Birth Group’s Specialized Register, and the Cochrane Central Register of Controlled Trials. We will use a three-step approach for the search strategy. An initial limited search of MEDLINE using PubMed will be undertaken followed by analysis of the text contained in the title and abstract, and of the index terms used to describe the articles. A second search using all identified keywords and index terms will then be undertaken across all included databases. We will search for unpublished studies in the grey literature through Open Grey, Google, Google Scholar, relevant conference abstracts, and Clinical Trials.gov. We will solicit unpublished reports from multilateral organizations (WHO, UNICEF, UNFPA, World Bank), bilateral agencies, and non-governmental organizations (NGOs) whose programs may include interventions for prevention of adverse pregnancy outcomes. Additionally, we will screen reference lists of included studies for potentially eligible studies. The search will be coordinated by a librarian and will not be limited by language or status of publication. The search strategy will first be formulated in PubMed and adapted for other search databases. Examples of terms and concepts that will be searched individually and in combination (Table 1) include ((“Labor, Obstetric”[Mesh] OR “Delivery, Obstetric”[Mesh]) AND “Transportation of Patients”[Mesh]) AND (“mortality”[Subheading] OR “mortality”[All Fields] OR “mortality”[Mesh])) AND (“Evaluation Studies as Topic”[Mesh] OR “Evaluation Studies”, OR “Program Evaluation”[Mesh] OR “Health Care Evaluation Mechanisms”[Mesh] OR “Health Care Quality, Access, and Evaluation”[Mesh] OR “Health Services Research”[Mesh] OR “Process Assessment Health Care”[Mesh] OR “Emergency Medicine”[MeSH]), “Parturition”[Mesh] “Pregnan*” OR “Labor” OR “Birth” OR “Labour” OR “Childbirth” OR “Deliver*”, “Low-Income Economies”, OR “Low-income country”, OR “Low-income countries”, OR “Lower-Middle-Income Economies”, OR “Resource-limited Setting*”, OR “Resource-poor settings”, OR “Poor countries” OR “Middle-income countries”, OR “Upper-Middle-Income Economies”, “transport*” OR “Transport* scheme” OR “Transport* system” OR “Voucher” OR “Loan” OR “Fund” OR “Revolving fund*” OR “Community financing”. All references will be managed using EndNote Version 7.

**Table 1:** Search strategy for identification of studies

No.	Search term
1	((“Labor, Obstetric”[Mesh] OR “Delivery, Obstetric”[Mesh]) AND “Transportation of Patients”[Mesh]) AND (“mortality”[Subheading] OR “mortality”[All Fields] OR “mortality”[Mesh])) AND (“Evaluation Studies as Topic”[Mesh] OR “Evaluation Studies”, OR “Program Evaluation”[Mesh] OR “Health Care Evaluation Mechanisms”[Mesh] OR “Health Care Quality, Access, and Evaluation”[Mesh] OR “Health Services Research”[Mesh] OR “Process Assessment Health Care”[Mesh] OR “Emergency Medicine”[MeSH]).
2	“Parturition”[Mesh] “Pregnan*” OR “Labor” OR “Birth” OR “Labour” OR “Childbirth” OR “Deliver*”
3	“Obstetric emergency”, or “Matern*near2 emergency”, OR “Emergency health service”, Or “Emergency health care”, OR “Emergency Obstetric Care”, OR “EmOC”, OR “Obstetric care”, OR “Pregnan*”.
3	“Pregnancy outcome”, OR “Adverse pregnancy outcome*”, OR “Adverse maternal outcome*”, OR “Adverse childbirth outcome*”, OR “Pregnancy complication*”, OR “Labour complicat*”, OR Labor complicat*”, OR “Birth AND “Complications”.
5	“Low-Income Economies”, OR Low-income country”, OR “Low-income countries”, OR “Lower-Middle-Income Economies”, OR “Resource-limited Setting*”, OR “Resource-poor settings”, OR “Poor countries” OR “Middle-income countries”, OR “Upper-Middle-Income Economies”.
6	“Health facility deliver*” OR “Hospital deliver*” OR “Clinic delivery” OR “Skilled attendant delivery”
7	“Access to care” OR “Maternal delay*”, OR “Access barrier*” OR “Geographic barrier”
8	“Intervention” OR “Interven*”, OR “Effect*”, OR “effective* OR “Impact” OR “Outcome*”, OR “Cost effect*”.
9	1-#8 AND “transport*” OR “Transport* scheme” OR “Transport* system” OR “Voucher” OR “Loan” OR “Fund” OR “Revolving fund*” OR “Community financing”.

### Study selection

A checklist (Table 2) of eligibility characteristics (type of study population, intervention, comparison, and study design (PICOS) will be used to screen studies for inclusion. First, two reviewers (EE and JC) will independently screen titles and abstracts of identified studies to assess their eligibility for inclusion. Second, EE and JC will screen the full texts from the first step to make a final determination regarding each study’s eligibility. Where there are uncertainties regarding eligibility, JE and MM will be consulted.

**Table 2:** Eligibility screening form

STUDY CHARACTERISTICS	YES	NO	UNCLEAR
1. STUDY DESIGN			
A) Randomized controlled trial			
B) Non-randomized comparative trial			
C) Cohort study			
D) Case-control (including studies with historical control group)			
E) Controlled before and after study			
F) Cross-sectional study with comparative analysis of subsets of exposed (cases) and unexposed (controls).			
2. STUDY PARTICIPANTS			
A) Women in the pre- to post-natal phase of pregnancy? (and/or)			
B) Infants associated with the pregnancy?			
3. STUDY INTERVENTION			
A) Transportation interventions for emergency obstetric care Provision of any of the following where none existed prior:			
• Any form of ground transportation (bicycle, motorcycle, oxcart, ambulance, etc.)			
• vouchers/subsidies for transportation			
• loans, or sets up a system of pooled funds			
4. CONTROL			
A) Did the control receive additional support for transportation?			
*5. OUTCOME MEASURES			
A) Were any of the following pregnancy-related outcomes reported?			
• Stillbirth and/or neonatal mortality rates			
• Maternal mortality rates,			
• Care-seeking, referral rates,			
• Reduced delay in access to care,			
• Facility delivery rates,			
• Costs-benefit analysis/cost-effectiveness			
6. DECISION			
A) Include?			
B) Exclude?			
C) UNCLEAR?			
7. COMMENTS /REASONS FOR EXCLUSION			

NOTE: A) include if all is “YES”. B) Exclude if 2A, 2B, 3A, 5A are “NO”. C) Otherwise “ UNCLEAR”

*Note that absence of outcome measure is not an exclusion criterion at this stage of eligibility screening; simply indicate outcomes assessed in each included study

### Assessment of risk of bias in included studies

Two reviewers (JE and HA) will assess the quality of included studies using the risk of bias criteria for randomized controlled trials, non-randomized controlled trials, and controlled before-and-after studies developed by the Cochrane Effective Practice and Organization of Care (EPOC) as an adaptation of the Cochrane Collaboration’s tool for assessment of risk of bias [45]. Disagreements between the two assessors will be resolved by discussion and consensus, with arbitration by a third reviewer as required. In line with EPOC guidelines, each criterion will be scored as “low risk,” “unclear risk,” or “high risk” (Table 3). A study will be considered to be of low risk if all EPOC risk of bias criteria are scored as “yes,” “unclear risk of bias” if one or more criteria are scored as “unclear,” and “high risk of bias” if the study is scored “no” on one or more key criteria. Finally, the GRADE approach will be used to determine the quality of evidence for the main outcomes. The GRADE is process for rating the quality of the best available evidence developed by the Grading of Recommendations, Assessment, Development and Evaluation (GRADE) Working Group [46]. As shown in Table 4, the GRADE approach uses results of four criteria to assess the quality of the body of evidence derived from a systematic review. Factors taken into consideration in the grading of evidence include study bias levels, consistency of results, directness of results, and precision of results.

**Table 3:** Methodological quality of included studies

Study ID (author/year)	Random sequence generation (selection bias)	Allocation concealment (selection bias)	Blinding (performance bias and detection bias)	Incomplete outcome data (attrition bias)	Selective reporting (reporting bias)	Other bias	Baseline outcome measurement similar?	Baseline characteristics similar?	Adequate protection against contamination?
ᅟ	ᅟ	ᅟ	ᅟ	ᅟ	ᅟ	ᅟ	ᅟ	ᅟ	ᅟ
ᅟ	ᅟ	ᅟ	ᅟ	ᅟ	ᅟ	ᅟ	ᅟ	ᅟ	ᅟ
ᅟ	ᅟ	ᅟ	ᅟ	ᅟ	ᅟ	ᅟ	ᅟ	ᅟ	ᅟ
ᅟ	ᅟ	ᅟ	ᅟ	ᅟ	ᅟ	ᅟ	ᅟ	ᅟ	ᅟ
ᅟ	ᅟ	ᅟ	ᅟ	ᅟ	ᅟ	ᅟ	ᅟ	ᅟ	ᅟ
ᅟ	ᅟ	ᅟ	ᅟ	ᅟ	ᅟ	ᅟ	ᅟ	ᅟ	ᅟ
ᅟ	ᅟ	ᅟ	ᅟ	ᅟ	ᅟ	ᅟ	ᅟ	ᅟ	ᅟ
ᅟ	ᅟ	ᅟ	ᅟ	ᅟ	ᅟ	ᅟ	ᅟ	ᅟ	ᅟ
ᅟ	ᅟ	ᅟ	ᅟ	ᅟ	ᅟ	ᅟ	ᅟ	ᅟ	ᅟ
ᅟ	ᅟ	ᅟ	ᅟ	ᅟ	ᅟ	ᅟ	ᅟ	ᅟ	ᅟ
ᅟ	ᅟ	ᅟ	ᅟ	ᅟ	ᅟ	ᅟ	ᅟ	ᅟ	ᅟ
ᅟ	ᅟ	ᅟ	ᅟ	ᅟ	ᅟ	ᅟ	ᅟ	ᅟ	ᅟ
ᅟ	ᅟ	ᅟ	ᅟ	ᅟ	ᅟ	ᅟ	ᅟ	ᅟ	ᅟ
ᅟ	ᅟ	ᅟ	ᅟ	ᅟ	ᅟ	ᅟ	ᅟ	ᅟ	ᅟ
ᅟ	ᅟ	ᅟ	ᅟ	ᅟ	ᅟ	ᅟ	ᅟ	ᅟ	ᅟ
ᅟ	ᅟ	ᅟ	ᅟ	ᅟ	ᅟ	ᅟ	ᅟ	ᅟ	ᅟ

**Table 4:** GRADE quality of evidence grades

Grade	Definition
High	We are very confident that the true effect lies close to that of the estimate of the effect.
Moderate	We are moderately confident in the effect estimate: The true effect is likely to be close to the estimate of the effect, but there is a possibility that it is substantially different.
Low	Our confidence in the effect estimate is limited: The true effect may be substantially different from the estimate of the effect.
Very low	We have very little confidence in the effect estimate: The true effect is likely to be substantially different from the estimate of effect.

### Data extraction

Two reviewers (IA and HA) will independently extract data from each eligible study, using the Cochrane Collaboration’s standard data extraction form [47]. We will resolve differences through discussion and consensus among all reviewers. We will extract data on study setting, design, participants’ characteristics, interventions, controls, and duration of follow-up. We will also extract data on sample size, age, and data collection methods. Where possible, we will obtain qualitative information on context and potential confounding. We will obtain data on cost if available. We will collect data on the primary outcome, mortality (stillbirth, maternal mortality, and neonatal mortality), and secondary outcomes, access to care (reduced delay accessing care, time taken to access care, or other measures of reduction in delay in accessing case), care-seeking behaviors, referral rates, facility delivery rates, cost, and cost-effectiveness of interventions. Where necessary, we will contact authors of included studies for additional information or missing data. This study will not require approval from the Internal Review Board. The proposed study is a secondary analysis of peer-reviewed publications. No human subjects will be directly involved.

### Data synthesis

We will perform quantitative data synthesis where studies are homogenous in characteristics and provide adequate outcome data for meta-analysis. Review Manager (version 5.3) will be utilized to perform fixed or random effect model meta-analysis. To detect statistical heterogeneity across included studies, we will conduct chi-square (*χ*^2^) and *I*-square (*I*^2^) tests. Significant heterogeneity will be determined by *χ*^2^ test with *p* value of < 0.1; heterogeneity will be adjudged to increase magnitude as *I*^2^ percentage point increases towards 100%. For sensitivity analysis, we will examine the residuals and chi-square components of estimates of effects and assess the effect of deleting each study in turn. In this case, where a statistically significant result depends on a single study, we will assess that study thoroughly with regard to sources of heterogeneity. We will perform meta-regression to adjust for potential confounding in multiple/mixed intervention studies, if we find sufficient number of eligible studies with these characteristics. Meta-analysis will not be performed if studies show marked heterogeneity (if *p* > 0.1). In addition, we will not perform meta-analysis if there are marked methodological variations, e.g., in types of modes of delivery of intervention. To synthesize quantitative data, we will undertake narrative synthesis, following the guidelines for conducting narrative synthesis in systematic reviews by Popay et al. [48].

## Discussion

Despite the availability of many emergency obstetric interventions in LMICs, approximately 60 million births still occur outside healthcare facilities and without skilled attendants [11], and LMICs continue to bear high burden of maternal and neonatal deaths [1, 2]. Pregnancy complications can be unpredictable, and many women in LMICs cannot access health facilities where life-saving care is available [49]. This review will highlight the extent to which interventions that promote access to and use of transportation increase women’s access to emergency obstetric care.

Emergency transportation primarily addresses one of the three delays that increase the risk of maternal deaths in LMICs, i.e., delay in reaching care [24]. Where data permits, this review will examine contextual factors that increase or hinder the effectiveness of transportation interventions in reducing adverse pregnancy outcomes in low- and middle-income countries. Such contextual factors may include health provider skills in detecting complications, decision to refer, and readiness of the receiving facility to provide needed care. It is hoped that the findings of this review will inform the global debate on access to emergency obstetric care in LMICs. Identification of cost-effective transportation interventions and contextual underpinnings can contribute to efforts to increase utilization of skilled obstetric care, and thus decrease the burden of maternal, neonatal, and infant mortality in LMICs.

## Additional file

Additional file 1: PRISMA-P (Preferred Reporting Items for Systematic Review and Meta-Analysis Protocols). (DOCX 19 kb)

## Abbreviations

LMICs: Low- and middle-income countries
MDG: Millennium Development Goal
MNCH: Maternal, Newborn and Child Health
